# Extravascular Lung Water and Effect on Oxygenation Assessed by Lung Ultrasound in Adult Cardiac Surgery

**DOI:** 10.7759/cureus.9953

**Published:** 2020-08-23

**Authors:** Francis Emperador, Sean R Bennett, Julia Gonzalez, Ahmed Saati, Basim S Alsaywid, Jose A Fernandez

**Affiliations:** 1 Critical Care, Great Ormond Street Hospital, London, GBR; 2 King Faisal Cardiac Center, Cardiac Anesthesiology, King Abdulaziz Medical City, Ministry of National Guard - Health Affairs, Jeddah, SAU; 3 Department of Surgery, King Abdulaziz Medical City, Ministry of National Guard - Health Affairs, Jeddah, SAU; 4 Research and Development Department, Clinical Epidemiology, King Abdulaziz University Hospital, Riyadh, SAU

**Keywords:** cardiac surgery, oxygenation, extravascular lung water, lung ultrasound (lus)

## Abstract

Introduction

The extravascular lung water content is determined by the use of lung ultrasound (LUS) which is represented as B-lines. The aim of this study was to investigate whether the LUS measurement of extravascular lung water was correlated to changes in oxygenation.

Methods

This prospective cohort study was comprised of 73 patients with an average age of 56 (range: 18 to 87 years) who underwent elective cardiac surgery using cardiopulmonary bypass. The LUS score was performed preoperatively, time zero (T0), at one hour (T1), and at 24 hours (T2) post-surgery. Additionally, arterial oxygen partial pressure and fraction of inspired oxygen (PaO_2_/FiO_2_) ratio were measured at each time and the time-to-extubation.

Results

A negative correlation was found between the LUS score and PaO_2_/FiO_2_ at T1 (p < 0.004). Extubation time and changes in the lung ultrasound score at T0 - T2 were positively correlated (p < 0.03). Plus, there was a positive correlation between fluid balance and lung ultrasound score at T2 (p < 0.03).

Conclusion

We found three significant correlations that support the use of LUS in cardiac surgery: 1) the more B-lines, the lower the oxygenation; 2) the more B-lines, the longer the period of ventilation; 3) the more B-lines, the more positive the fluid balance. LUS is a non-invasive bedside investigation that can be used to judge extravascular lung water, providing useful information in the management of patient oxygenation, fluid balance, and extubation.

## Introduction

Lung ultrasound (LUS) has received much attention in the last decade due to its use in many areas: critical care, emergency medicine, trauma surgery, and more recently, paediatric and adult cardiac surgery, in which it offers an important tool for monitoring and diagnostic purposes [1-2].

LUS is a bedside tool that is free of radiation, reproducible, and cost-effective [1]. LUS can detect excess extravascular lung water (EVLW) and is a predictor of pulmonary oedema. EVLW is the amount of fluid that accumulates in the interstitial and alveolar spaces. It is represented by B-lines, which are artifacts that appear on LUS [2].

Most adult patients having cardiac surgery using cardiopulmonary bypass (CPB) have a positive fluid balance postoperatively. Early extubation (less than four hours) has become normal for routine cardiac surgery. This requires attention to body temperature, analgesia, and bleeding but also a fluid balance that avoids pulmonary oedema or hypoxia as positive pressure ventilation is discontinued.

Pulmonary complications relating to excess lung water include poor oxygenation and reduced compliance resulting in prolonged ventilation. Knowing the preoperative state of the EVLW for the patient and optimizing the perioperative care can be crucial for improving the outcomes after cardiac surgery. However, there are currently no guidelines for the use of LUS post-cardiac surgery and few data related to this topic.

The aim of this study was to assess EVLW before and after cardiac surgery by scoring B-lines using LUS. The primary outcomes were to assess the relationship between B-lines and the effect on oxygenation and time of extubation (Extub T).

Secondary outcomes were the influence of CPB time, aortic cross-clamp (ACC) time, and fluid balance at one hour (T1) and 24 hours postoperatively (T2) on oxygenation. Also recorded was the presence of pleural effusion (PE) and consolidation.

## Materials and methods

The study was designed as a prospective cohort study in elective cardiac surgery using CPB. The study was performed at the King Abdulaziz Medical City in the National Guard, King Faisal Cardiac Center between March 2018 and March 2019. The protocol was approved by the Institutional Review Board at the King Abdullah International Medical Research Center, #RYD-18-417780-17652. Written consent was obtained from all patients prior to surgery.

The inclusion criteria were patients older than 18 years who were scheduled for elective cardiac surgery using CPB. Patients with pulmonary fibrosis and chronic obstructive lung disease were included. The exclusion criteria were emergency procedures.

The patient characteristics and baseline clinical data were collected for every patient, including age, weight, height, diagnosis, previous pulmonary pathology, logistic European System for Cardiac Operative Risk Evaluation (euroSCORE) [3], and type of surgery (Table 1).

**Table 1:** Patient Characteristics for the 73 Patients Who Completed the Study CABG: coronary artery bypass graft

Age (years), mean (range)	56 (18 - 87)
Gender (male/female %)	70/30
Body mass index mean kg/mt^2 ^(range)	28.6 (16.5 - 51.8)
EuroScore mean %	2.13
Previous pulmonary pathology %	22
Type of surgery % CABG	62
Valve only	28
Valve and CABG	7
Excision of tumour	1

Standard monitoring for cardiac surgery was performed, including electrocardiography, oxygen saturation, invasive blood pressure, central venous pressure, cerebral oximetry, and nasopharyngeal temperature. Patients were premedicated with 1 mg intravenous (IV) midazolam prior to induction. Anaesthesia consisted of fentanyl 1-2 μg/kg, propofol 2 mg/kg, and rocuronium 1.2 mg/kg. Patients received boluses of IV phenylephrine or IV nitroglycerine as required. General anaesthesia was maintained with sevofluorane at 0.7 to 1.0 minimal alveolar concentration. Rocuronium, 0.6 mg/kg bolus, and fentanyl, 1 μg/kg, was repeated during surgery as required. On CPB, anaesthesia was sevoflurane 1% - 2%. At the end of the CPB and removal of the aortic clamp, the lungs were recruited with a continuous positive airway pressure (CPAP) of 30 cmH_2_O for 5 - 10 seconds. Vasoactive drugs were guided by the patient’s haemodynamic parameters: norepinephrine 0.01 - 0.05 μg/kg/min, nitroglycerin 1 - 4 μg/kg/min, epinephrine 0.01 - 0.05 μg/kg/min, or milrinone 0.2 - 0.5 μg/kg/min.

Every patient had a standard fluid protocol during anaesthesia, CPB, and intensive care unit (ICU) stay according to body weight, plus a bolus of IV fluids if indicated. Fluid during CPB was counted in the balance calculation: (+) crystalloids/colloids/blood products; (-) bleeding/insensible loss/urine, and in the Intensive Care Unit (ICU), drains. Data was collected hourly on our database.

Blood transfusion followed the hospital guidelines for Perioperative Blood Transfusion and Blood Conservation in Cardiac Surgery using the Society of Thoracic Surgeons and the Society of Cardiovascular Anesthesiologists Clinical Practice Guideline [4].

The ventilation strategy was:

Pre-bypass: volume control respiratory rate (RR) 10 - 12 per minute (keeping CO_2_ between 35 - 45 mmHg), tidal volume: 4 - 6 ml/kg, positive end expiratory pressure (PEEP) 0, fraction of inspired oxygen (FiO_2_) 0.5, and inspiratory:expiratory (I:E) 1:2.

Post-bypass: volume control RR 10 - 12 (keeping CO_2_ between 35 - 45 mmHg), tidal volume: 4 - 6 ml/kg, PEEP 0, FiO_2_ 0.5, and I:E 1:2.

ICU: volume: 4 - 6 ml/kg and PEEP 5+ according to FiO_2_. O_2_ started at 50% until the first arterial blood gas sample and adjusted.

Weaning protocol: Patients were not routinely sedated. Patients received analgesia by fentanyl infusion and paracetamol. When the patient started to respond, they were put onto synchronized intermittent mandatory ventilation (SIMV) and then pressure support prior to extubation. FiO_2_: < 0.4, PEEP 5, pressure support: 10. A chest x-ray was obtained before extubation.

**Figure 1:**
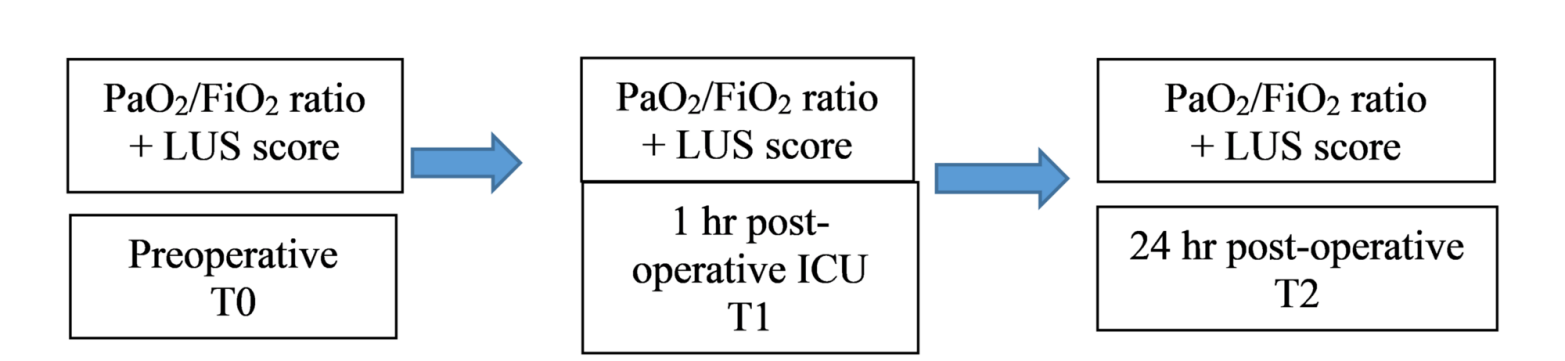
Timeline representing the study protocol FiO_2_: fractional inspired oxygen; ICU: intensive care unit; LUS: lung ultrasound; PaO_2_: partial pressure arterial oxygen; T0: awake immediately pre-surgery; T1: one-hour post-surgery; T2: 24 hours post-surgery

The LUS examination was performed using a Philips CX50 and phased array probe 2 - 4 MHZ with a lung ultrasound preset (Koninklijke Philips N.V., Amsterdam, Netherlands). The LUS score was recorded pre-surgery (awake) (T0), one-hour post-surgery (T1), and 24 hours post-surgery (T2), as well as oxygenation (Figure 1).

**Figure 2:**
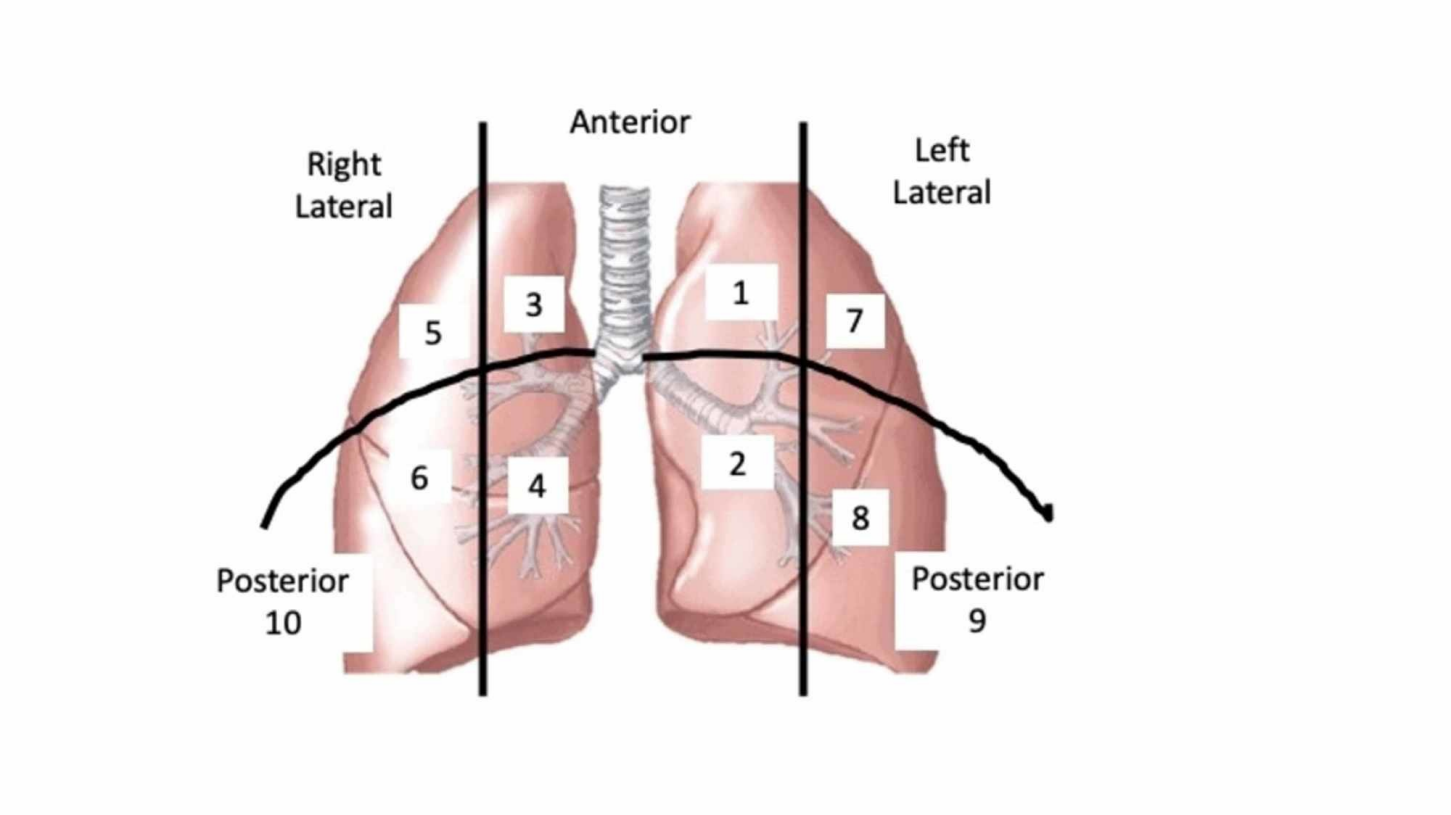
Anatomical areas on which the lung ultrasound was performed. Ten areas were examined (1 - 10). The lung ultrasound score was the total of the scores for the 10 areas. Figure 2 shows the corresponding numbers for the right and left sides of the thorax.

The ultrasound was performed by an anaesthetist who had experience in LUS. Patients were scanned in the supine position according to the method described by Volpicelli et al. [5]. We included a posterior scan area in addition so that a better appraisal of effusion and consolidation could be made. This may have increased the LUS score, but the conditions were the same for all patients. Thus, we had 10 anatomical sites that were assessed by LUS (Figure 2).

**Figure 3:**
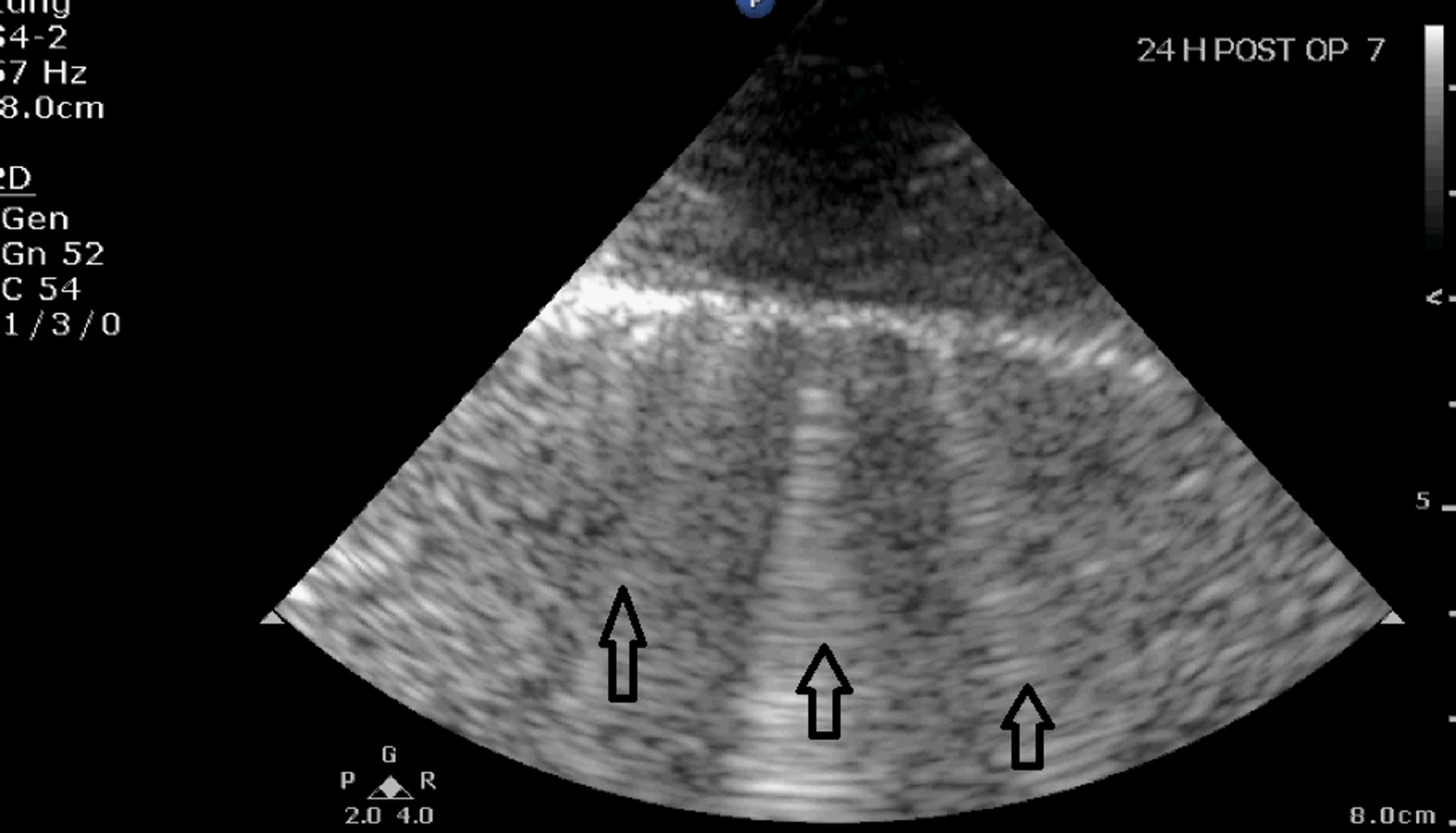
Lung ultrasound image showing B-lines (arrows) 24 hours after cardiac surgery

**Figure 4:**
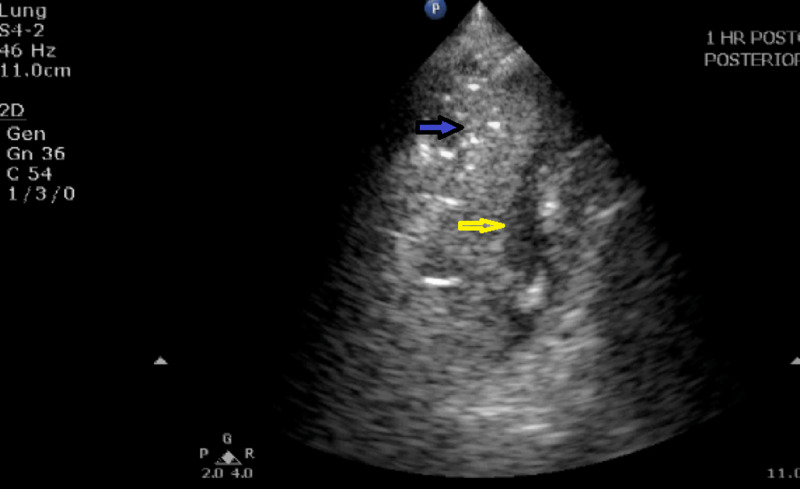
Lung ultrasound image showing consolidation and pleural effusion at T1 The figure shows consolidation (blue arrow) and a small pleural effusion (yellow arrow) at T1 (one hour post-surgery).

The LUS score was calculated according to the number of B-lines and the presence of a pleural effusion and/or consolidation which was recorded as ‘yes’ or ‘no’ (Table 2). Images of B-lines, pleural effusion, and consolidation are shown in Figures 3, 4.

**Table 2:** Scoring System for B-Lines Consolidation and pleural effusion were recorded as Y-yes or N-no

Ultrasound Profile	Number	Score
A-lines	Normal profile 1 - 2 lines	0
B-lines	Normal profile 1 - 2 lines	0
B-lines	3 - 5 lines	1
B-lines	> 5 lines	2
Pleural effusion		Y/N
Consolidation		Y/N

Statistics

Sample Size

At the time of the protocol, there were no previous studies investigating LUS for the assessment of EVLW in adult cardiac surgery. Our sample size was calculated for our primary outcome which was the assessment of B-lines and fluid overload from previous data obtained in 61 paediatric patients undergoing cardiac surgery [2]. To detect a correlation of 0.3, with 5% significance and power of 80%, we aimed to recruit 82 patients. However, after 50 patients were studied, an interim analysis allowed us to adjust this figure to 78. It was a non-randomized study in which we used non-probability, consequent sampling tests.

Analytical Methods

All data are expressed as mean +/- standard deviation (SD) or median. The primary outcomes were evaluated using the chi-square test, Spearman’s and Pearson correlation analysis reported with a level of significance of p < 0.05, two-tailed. The secondary outcomes were evaluated using the Student’s test and chi-square test for the continuous and categorical variables, respectively. Simple descriptive and correlation analyses were performed in IBM Statistical Package for Social Sciences (SPSS), version 23 (IBM SPSS Statistics, Armonk, NY) and the R Project for Statistical Computing, version 3.6.2 (http://www.r-project.org/).

## Results

**Figure 5:**
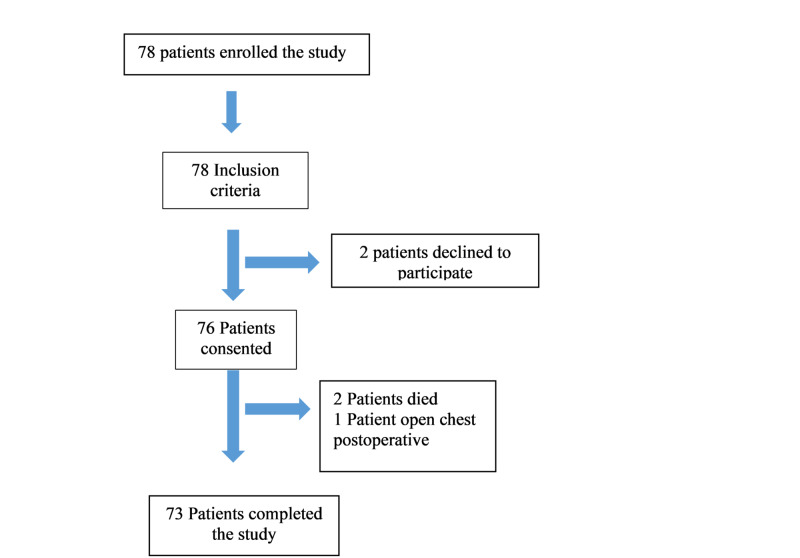
Consolidated Standards of Reporting Trials (CONSORT) flow diagram

Figure 5 shows the selection of participants in the study.

Table 3 shows the correlation of the LUS score and oxygenation at the three study time points, T0, T1, and T2. Of note, there was a significant negative correlation with the change in LUS score at T1, and changes in PaO_2_/FiO_2_ (p < 0.004). However, the change in the LUS score at T2 and PaO_2_/FiO_2_ was not statistically significant (p < 0.81). There was no significant statistical correlation between ACC and CPB times with the LUS score. There is a progressive increase in both consolidation and pleural effusion in the first 24 hours.

**Table 3:** Correlation Between Oxygenation, Cardiopulmonary Bypass Times, and Lung Ultrasound Scores at the Three Study Time Points and the Incidence of Consolidation and Pleural Effusion *Significant result by Spearman correlation. **Procedure not performed at T0 CPB: cardiopulmonary bypass; FiO_2_: fractional oxygen concentration; LUS: lung ultrasound; PaO_2_: partial pressure arterial oxygen; T0: awake immediately pre-surgery

	LUS score at T0 = 4.36	LUS score at T1 = 7.04	LUS score at T2 = 7.83
PaO_2_/FiO_2 _	0.361	-0.004*	-0.81
Aortic clamp time	**	0.59	0.66
CPB time	**	0.29	0.90
Incidence of consolidation (%)	11	39	50
Incidence of pleural effusion (%)	7.7	15	35

Table 4 shows the influence of certain procedures on changes in LUS from baseline. One finding relating to our primary objective was a statistically significant positive correlation of increasing LUS score between T0 and T2 and Extub T (p < 0.03). Looking at the secondary objectives, a higher positive fluid balance at one-hour post-surgery resulted in a significant increase in the LUS score between T0 and T2 (p < 0.03).

**Table 4:** Recorded Procedural Data That May Have Caused Changes in the Lung Ultrasound Score From Baseline *Significant result by Pearson correlation CPB: cardiopulmonary bypass; delta T0 to T1: difference in lung ultrasound score from preoperatively (TO) to one-hour after surgery (T1); delta T0 to T2: difference in lung ultrasound score from preoperatively to 24 hours after surgery (T2); LUS: lung ultrasound

	LUS score delta T0 to T1 = +2.68	LUS score delta T0 to T2 = +3.47
Extubation time	0.08	0.03*
Aortic clamp time	0.47	0.97
CPB time	0.80	0.76
Fluid balance after surgery (T1)	0.11	0.03*
Fluid balance after 24 hours (T2)	0.07	0.36

## Discussion

Our goal was to see if it was practical to use LUS in cardiac surgery to show LUS signs of EVLW and demonstrate a correlation between LUS signs and oxygenation. The principle LUS sign was B-lines which were scored. Comparing preoperative and postoperative LUS scores and PaO_2_/FiO_2_, we found a significant negative correlation between B-lines and oxygenation. This is in agreement with a study of two groups of patients undergoing coarctation repair and treated with furosemide in which lung oedema and B-lines, scored by LUS, were less in the treated group [6]. Picano and Pellikka discussed that LUS could detect subclinical forms of pulmonary edema represented as B-lines [7]. Volpicelli et al. added that there was not always a correlation between elevated pulmonary artery occlusion pressure and the finding of a B-pattern with LUS [8]. LUS did have better specificity in the detection of a B-pattern for increased EVLW than by the Pulse Index Contour Continuous Cardiac Output (PiCCO) device (Pulsion Medical Systems, Feldkirchen, Germany). They noted that B-lines identifying EVLW could have a cardiogenic or non-cardiogenic mechanism. 

An increase in B-lines caused worsening oxygenation as measured by PaO_2_/FiO_2_ and was associated with longer periods of ventilation. This finding is in agreement with a randomized control trial where LUS was used in congenital heart surgery in which two groups were compared: one with conventional ultrafiltration and the control which did not receive ultrafiltration [9]. At the end of the surgery, there was a negative correlation with PaO_2_/FiO_2_ and LUS score. Another study was able to use LUS to identify patients with increased EVLW, demonstrated by B-lines and a decreased of PaO_2_/FiO_2_, which was correctable by dialysis [10]. Similar results were seen in other studies performed in adult patients undergoing haemodialysis, both of which showed that removal of volume in patients undergoing chronic dialysis resulted in a reduction of B-lines [11-12]. This supports our finding of a high LUS score and worse oxygenation. Plus, an increase in LUS score (T0 to T2) was positively correlated with fluid management. This positive correlation is the most likely reason for the increased postoperative LUS scores being associated with longer time-to-extubation.

Despite most patients being extubated by 24 hours, they actually had slightly lower PaO_2_/FiO_2_ T2 = 328 versus T1 = 331. The lung LUS score at 24 hours was higher, explaining the deterioration in oxygenation. Thus, patient fluid management was again helped in the later postoperative period by using a technique that involved no radiation.

Do these relatively novel uses of LUS help the patient’s management by determining the EVLW status prior to surgery? Can this guide fluid management during surgery and in the ICU? A review article has mentioned the impact of LUS as a monitoring technique for cardiothoracic and vascular patients. The author emphasizes that LUS could improve perioperative outcomes [13]. More recently, an expert consensus highlighted the importance of the use of LUS in patients undergoing cardiac surgery with CPB who also suffer from coronavirus disease 2019 (COVID-19) [14].

While performing LUS, both pleural effusions and consolidation are seen. These secondary outcomes increased significantly postoperatively. Our incidence of postoperative pleural effusion of 34% was similar to the study by Touw et al. which found an incidence of 33% using LUS and 28% detected by chest x-ray after cardiac surgery [15].

Another advantage of LUS was the ability to detect and differentiate consolidation from the pleural fluid which is difficult with a chest x-ray. This was demonstrated by a prospective observational study where LUS was compared with a chest x-ray to diagnose postoperative pulmonary complications following cardiothoracic surgery [15]. On early admission to ICU, they found consolidation in 7.9% with LUS versus 0.0% with a chest x-ray. Overall, they found more postoperative pulmonary complications using LUS than a chest x-ray. The accuracy of LUS compared with chest x-ray impacts clinically on postoperative chest care. Alsaddique et al., on average, found 83 new respiratory abnormalities at three time points (after extubation, after removal of chest drains, and the day after surgery) [16]. The diagnosis, compared to chest x-ray, was changed in 52% of clinical episodes.

Study limitations

The anaesthetists who performed the LUS were not blinded, which may have influenced the outcomes measured. Assessment of fluid balance was well-recorded but was not standardized for all conditions, so fluid boluses were at the discretion of the anaesthetist and intensivist. Regarding physical limitations, it was more difficult to perform the LUS on obese patients due to the thickness of the chest wall. Also, when the surgery involved the dissection of the left mammary artery, the upper left superior area of the lung was more challenging to assess because of subcutaneous emphysema. Postoperatively, the presence of dressings and drains also caused some variation of probe position to obtain a good ultrasound view. We did not have a gold standard to compare with the LUS.

## Conclusions

This study describes a portable, non-invasive alternative to chest x-ray in cardiac surgery to assess EVLW. Our results indicate that lung ultrasound is a feasible bedside, non-invasive investigation that it can be used to judge EVLW. EVLW affects oxygenation and thus provides valuable information regarding fluid balance, extubation, and enhances decision-making. Other methods, such as PiCCO, are more invasive and a comparison is warranted. We believe that this LUS method for the assessment of EVLW helps to determine the fluid management in the paradigm of the hypoxic patient which can have many causes.
